# Activation of Emergency Department Stroke Protocol by Emergency Medical Services: A Retrospective Cross-Sectional Study

**DOI:** 10.3390/jcm14145041

**Published:** 2025-07-16

**Authors:** Noa Arad, Roman Sonkin, Eli Jaffe, Gal Pachys, Refael Strugo, Shiran Avisar, Aya Cohen, Ronen Levite, Itzhak Kimiagar, Shani Avnery Kalmanovich, Hunter Sandler, Ethan Feig, Nadya Kagansky, Daniel Trotzky

**Affiliations:** 1 Department of Emergency Medicine, Shamir (Assaf Harofeh) Medical Center, Zerifin 70300, Israel; 2 Faculty of Medical and Health Sciences, Tel Aviv University, Tel-Aviv 6997801, Israel; 3 Community Division, Magen David Adom, Or-Yehuda 6021805, Israel; 4 Department of Internal Medicine E, Soroka Medical Center, Beer-Sheva 84101, Israel; 5 Faculty of Health Sciences, Ben-Gurion University of the Negev, Beer-Sheva 8410501, Israel; 6 Division of Emergency Medicine, Shaare Zedek Medical Center, Jerusalem 9103102, Israel; 7 The School of Medicine, The Hebrew University, Jerusalem 9112102, Israel; 8 Medical Management, Sourasky (Ichilov) Medical Center, Tel-Aviv 6423906, Israel; 9 Department of Neurology, Shamir (Assaf Harofeh) Medical Center, Zerifin 70300, Israel; 10 Department of Interventional Neuroradiology, Shamir (Assaf Harofeh) Medical Center, Zerifin 70300, Israel; 11 New York Medical College (NYMC), MetHarlem, New York, NY 10037, USA; 12 Shmuel Harofe Geriatric Medical Center, Be’er Ya’akov 70350, Israel; 13 Department of Epidemiology and Preventive Medicine, School of Public Health, Gray Faculty of Medical & Health Sciences, Tel Aviv University, Tel Aviv 6997801, Israel

**Keywords:** stroke, prehospital assessment, field triage, emergency medical services, stroke protocol, emergency department

## Abstract

**Background/Objectives**: Early diagnosis of stroke is crucial for effective treatment with tissue plasminogen activator (tPA) and endovascular thrombectomy. Emergency medical services (EMSs) screening and the early activation of emergency department (ED) stroke protocols reduce treatment times and improve patient outcomes. This study aims to validate ED stroke protocol activation by EMSs in a large stroke center. **Methods**: This retrospective cross-sectional study was conducted at Magen David Adom and Shamir Medical Center between 1 January 2019 and 31 December 2019. Data were categorized into patients suspected by EMSs of having a stroke and those not suspected by EMSs but diagnosed as having a stroke in the ED. The primary outcome was the accuracy of EMSs in activating ED stroke protocols. **Results**: In this study, there were 23,061 patients, of which 11,841 (51.9%) were females. The mean age was 61.4 (SD = 22.72) years old. EMSs suspected 743 (3.22%) patients were having a stroke. In 587 (79%), EMSs activated ED stroke protocols. There were 88 cases where strokes were diagnosed in the ED when EMSs did not suspect a stroke. The overall EMSs negative predictive value (NPV) was 100% while the positive predictive value (PPV) was 20%. **Conclusions**: While Israeli EMSs over-activate the ED stroke protocol, stroke patients are almost never missed, achieving the goal of prehospital stroke screening. To prevent resource waste, all involved teams should be notified, and the actual activation of the stroke protocol should be carried out by an ED physician upon patient arrival. Communication between all levels regarding stroke protocol should also be increased to decrease the time to treatment.

## 1. Introduction

Common causes of stroke are intraparenchymal hemorrhage, cerebral infarction, subarachnoid hemorrhage, and other vascular events that cause a decrease in cerebral perfusion [1]. Vulnerable areas in the brain, such as the hippocampus, cerebellum, neocortex, and watershed areas (poorly perfused areas), can be damaged in five minutes by complete loss of perfusion, while other organs like the heart or kidney experience necrosis after 20–40 min [2]. Risk factors associated with strokes include increased age, male sex, systolic blood pressure (SBP), current smoking status, and diabetes mellitus (DM) [3].

The morbidity and mortality from strokes pose major declines in the quality of life for patients and place a great burden on their families and the healthcare systems. Over 80% of stroke patients experience acute hemiparesis in the first six months after a stroke, and more than 40% of stroke victims experience these symptoms chronically. Deficits in somatic sensations such as touch, temperature, pain, and proprioception are present in 11–85% of stroke victims [4].

The two main interventional stroke treatments are intravenous thrombolysis, which must be administered within 4.5 h of the stroke, and endovascular thrombectomy, which must be administered within 6 h, both with the goal to re-establish perfusion to the infarcted area [5]. Treatment time for strokes has been shown to be critical for a patient’s prognosis [1]. The saying “time is brain” exemplifies that every minute the brain does not receive oxygen, 1.9 million neurons die [6]. The administration of tissue plasminogen activator (tPA) in the first 90 min after a stroke reduces mortality, lowers the chances of symptomatic intracranial hemorrhage, and results in an earlier discharge time when compared to when tPA was administered within 181–270 min after stroke onset [7]. This has led to advanced healthcare systems implementing both internal and cross-organizational protocols for the screening and activation of proper systems to recognize stroke patients and shorten the time to treatment [7]. In some regions, like rural Alberta, mobile stroke units have been implemented to further expedite the administration of tPA and decrease neuron loss [8].

Advanced EMS systems utilize tests to screen for strokes and inform hospitals of incoming stroke patients. Common screening tests used by EMSs worldwide are the Field Assessment Stroke Triage for Emergency Destination (FAST-ED), Rapid Arterial Occlusion Evaluation (RACE), the Los Angeles Prehospital Stroke Screening (LAPSS) test, the PreHospital Ambulance Stroke Test (PreHAST) and others [9,10,11]. The FAST-ED exam evaluates facial weakness (scored 0–3), arm weakness (scored 0–4), slurred speech (scored 0–3), the importance of time, eye deviation (scored 0–2), and denial/neglect (scored 0–2) [1]. Research has shown that a FAST-ED exam with a score 4 or greater has a sensitivity of 60%, specificity of 89%, positive predictive value of 72%, and negative predictive value of 82% [9]. The RACE examination evaluates a patient for facial palsy (scored 0–2), arm motor function (0–2), leg motor function (0–2), gaze (0–1), and aphasia or agnosia (0–2). A RACE exam with a score 5 or greater had a sensitivity of 85%, specificity of 68%, positive predictive value of 42%, and negative predictive value of 94% [12]. The LAPSS examines a patient’s age, blood glucose levels, duration of symptoms, history of seizures, and motor asymmetry to diagnose strokes [3]. Research on the accuracy of the LAPSS exam demonstrated a sensitivity of 91%, specificity of 97%, PPV of 86%, and NPV of 98% [13]. The PreHAST test evaluates a patient’s ability to follow commands (0–2), eye position (0–2), visual field (0–2), facial palsy, arm paresis (0–2), leg paresis (0–2), sensory (0–2), and speech/language (0–2). The sensitivity of PreHAST was found to be 100% with a specificity of 40% for stroke or transient ischemic attack (TIA) when a positive score in any PreHAST item (PreHAST score 1–19 points) was found after prehospital assessment in patients with suspected stroke. The positive and negative predictive values were 50% and 100% for a positive PreHAST score [11].

Several studies assessed the overall validity of prehospital stroke screening and activation of stroke protocols in the ED upon EMS notification. In these studies, sensitivity varied between 60 and 80%, while specificity varied between 23.9 and 48%. The PPV and NPV ranged between 40 and 47.1% and 58.5–61%, respectively [9,14,15].

The protocols of Israel’s national emergency medical services organization, Magen David Adom (MDA), define a suspected stroke as any new neurologic deficit occurring in the 12 h prior to ambulance arrival and warrants stroke screening based on FAST-ED [9]. Upon positive screening, the stroke center’s ED is notified through the dispatch center. The threshold for ED stroke protocol activation is low to minimize the number of stroke patients missed. However, to reduce unnecessary consumption of valuable resources, a working cooperation was established between MDA and EDs to allow for the immediate assessment of incoming patients with a suspected stroke by an emergency medicine physician to either continue the stroke protocol or move the patient into the regular ED reception flow. Studies have shown that the evaluation of a stroke patient upon arrival to the ED by an emergency physician decreased door-to-needle time and onset-to-treatment time and increased accurate diagnosis. Using the pre-stroke protocol, emergency physicians can treat stroke patients quickly and efficiently with cooperation between neurologists, radiologists, and other emergency physicians [16].

The goal of this study was to assess the accuracy of stroke protocol activation by the EMSs when compared to the final diagnosis by the ED. 

## 2. Materials and Methods

### 2.1. Setting

**Figure 1:**
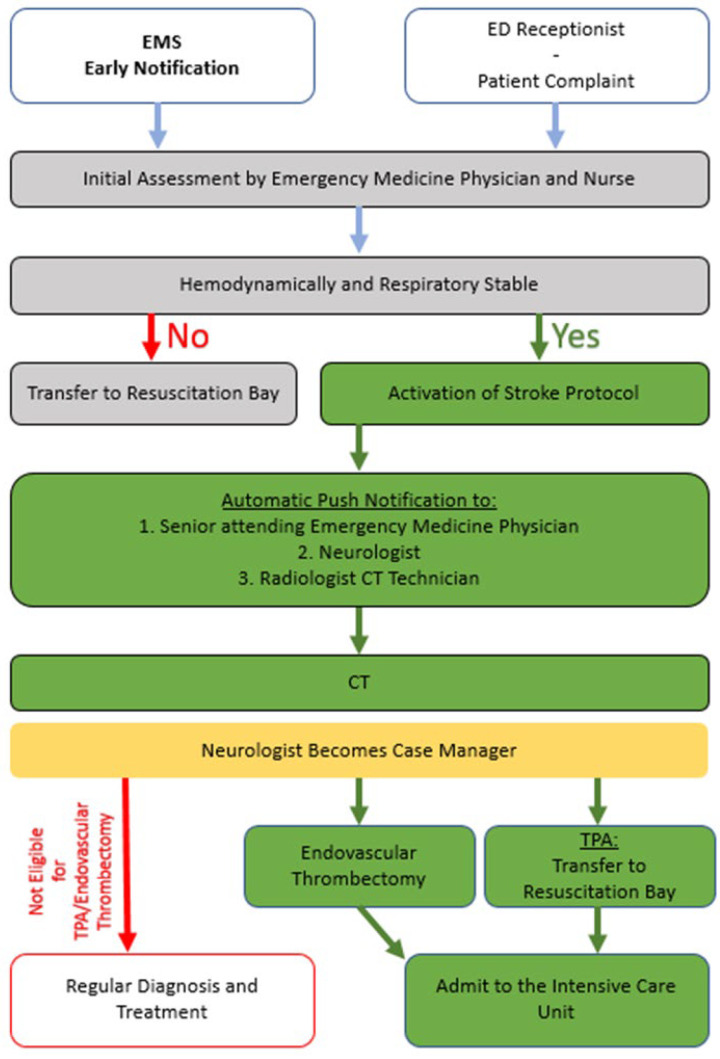
Shamir Medical Center stroke protocol.

This retrospective observational study was conducted at the Shamir Medical Center (SMC) and MDA between 1 January 2019 to 31 December 2019. SMC is an 848-bed academic level 1 Medical Center and Stroke Center that provides care for over 1 million residents of Israel’s central region. The Emergency Medicine Department is the fourth largest in Israel, treating about 160,000 patients annually. SMC diagnoses are based on the ICD 9 manual as is accepted in Israel. The SMC stroke protocol begins with suspicion of stroke by either a primary care physician, notification by EMSs, previously admitted patient transferred to the ED by a neurologist referral, or based on the ED receptionist suspicion from using the stroke symptom checklist (including disturbance of speech, facial expression, limb weakness, visual disturbance, and symptoms beginning in the past 4.5 h). The suspicion of a stroke initiates the placement of the patient on a monitored or intensive care bed in the ED. Automatic push notifications are sent to a neurologist, emergency medicine physician, and radiologist, and an initial workup is performed by a nurse. Both a neurologist and emergency medicine physician examine the patient for neurological signs and symptoms and comorbidities. After determining if the patient is experiencing a stroke, the type of treatment (tPA or endovascular thrombectomy) is decided upon, and treatment is initiated (Figure 1).

MDA is Israel’s national emergency medical services organization. MDA operates through a four-tiered response system; the first two tiers are two types of first responders, while the last are basic life support (BLS) and advanced life support (ALS) ambulances. All types of responses are coordinated by the MDA command-and-control systems connected to a dedicated application installed on responders’ smartphones and on the response vehicles’ tablet devices. BLS teams include an emergency medical technician (EMT) who is both the team leader and ambulance driver, along with another team member, either an EMT or first aid provider. They, in turn, operate a general ambulance equipped to provide simple medical treatment and BLS before transporting the patient to the hospital, if necessary. EMTs in Israel are proficient in BLS, can operate automated external defibrillators, prepare medications for use by paramedics, and assist in endotracheal intubation. The ALS teams include a paramedic team leader alongside an ambulance driver who is either an EMT or a paramedic. These are reserved for cases of higher severity. As part of their training, paramedics undertake courses in ALS, prehospital trauma life support, and pediatric advanced life support. Many paramedics elect to undertake a bachelor’s degree [17,18,19,20].

### 2.2. Study Design and Participants

This study followed the Reporting of studies Conducted using Observational Routinely collected health Data (RECORD) [21], and Standards for Reporting Diagnostic Accuracy (STARD) guidelines [22]. Clinical data about patients evacuated by MDA to SMC ED was retrospectively analyzed. The data was extracted from MDA and SMC databases and included all patients over the age of 18 who were evacuated by MDA ambulances to SMC during the studied period. Upon linking, MDA and SMC tables data was unidentified. The study was approved by the SMC institutional review board (IRB), approval number ASAF-0229-20, and the MDA scientific board. The IRB waived patient approval as the data were unidentified.

The EMS diagnosis of acute stroke includes not only stroke and stroke equivalents, namely acute ischemic stroke, transient ischemic attack, hemorrhagic stroke, and intracranial hemorrhage, but also any other illness that causes neurologic deficits. This results in a broader differential diagnosis than the hospital and results in more activation of ED stroke protocols by EMSs. The aim of the study is to evaluate the alignment between MDA teams’ decision to activate the stroke protocol and the decision made by the emergency medicine physician.

Data containing all patients that arrived at the ED by an MDA ambulance were extracted from the SMC database for a total of 23,061 patients, and two types of datasets were created. One contained data on patients for whom the EMSs activated stroke protocol due to suspicion of a stroke or a stroke equivalent. EMSs used a positive FAST-ED test or other clinical indications to suspect a stroke. The other dataset contained patients who were not suspected by the EMSs of having experienced a stroke or a stroke equivalent, and thus, stroke protocol was not initiated. Both datasets were compared with the final diagnosis from the ED. The sensitivity, specificity, PPV, and NPV of the activation of stroke protocol by MDA were calculated by comparing the MDA diagnosis to the final diagnosis of the emergency physician at SMC. This was calculated by comparing the activation of stroke protocol by BLS ambulances, ALS ambulances, and both BLS and ALS against the final ED diagnosis.

Cases when a diagnosis was not achieved were not included in the extracted data from the beginning. These cases are composed of patients who left the ED of their own volition or left without notice and refused to return.

Diagnoses were grouped into the following twelve categories: ischemic stroke, hemorrhagic stroke, TIA, intracranial hemorrhage, vascular, neurologic (other), other, trauma, toxicologic, cardiac, and psychiatric.

**Figure 2:**
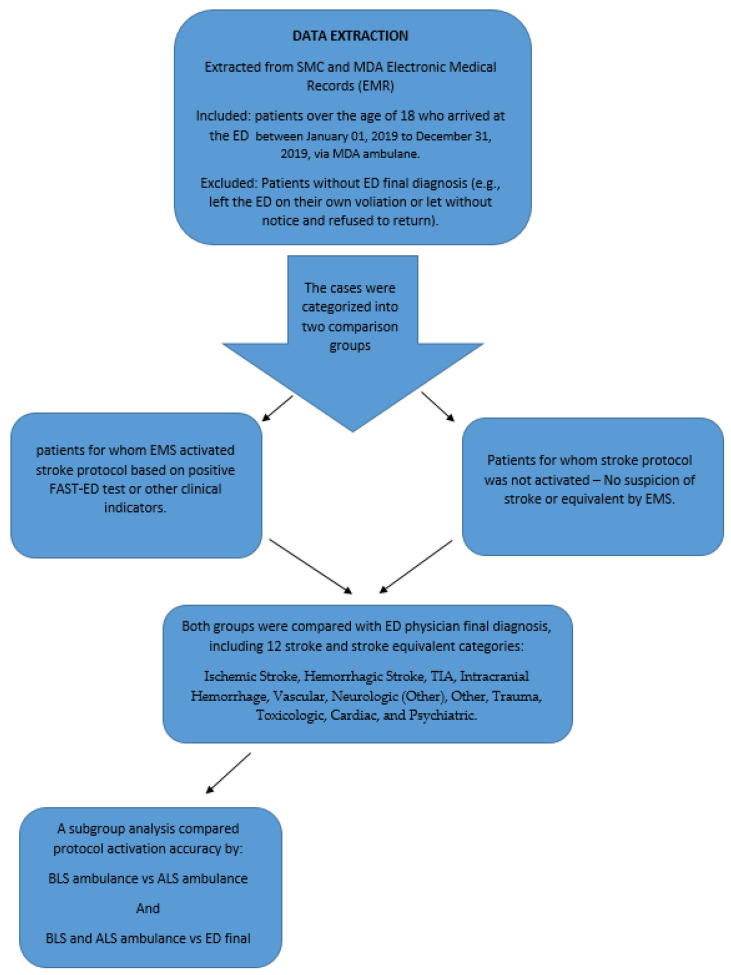
Study Flowchart.

Comparisons were run for BLS ambulances, ALS ambulances, cases when both types of responses were dispatched, and for EMSs as a whole (Figure 2).

### 2.3. Statistical Analysis

Chi2 was used to compare EMS and ED diagnoses and a *p*-value of less than 0.05 was considered significant. R (version 4.1.3) was used for statistical analysis using the caret package [23,24].

## 3. Results

A total of 23,061 patients were transported by MDA to the SMC ED, of which 11,841 (51.34%) were females. Of all the patients that arrived by MDA ambulance to the ED, 7657 (33.20%) were admitted to the hospital. The mean age was 61.4 (SD = 22.72).

**Figure 3:**
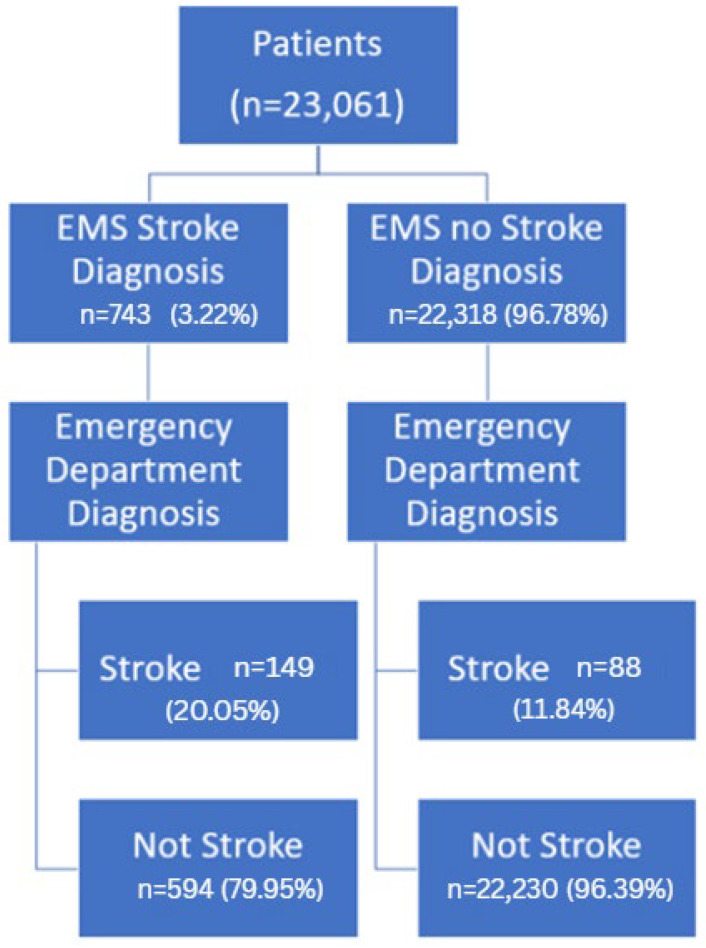
Flowchart of stroke diagnosis by EMSs vs. ED.

EMSs suspected 743 (3.22%) patients of having a stroke. Of these 743 patients suspected by EMSs, the ED confirmed the stroke diagnosis in 149 cases, and 594 cases were determined to not have experienced a stroke. EMSs did not suspect a stroke in 22,318 cases, of which 88 patients were diagnosed with a stroke by the ED and 22,380 patients were confirmed to have experienced a stroke. These results are shown in Figure 3.

EMSs performed a FAST-ED test on 613 of the 743 patients suspected of having a stroke (82.5%). Of the 130 (17.5%) cases where FAST-ED was not conducted, due to a lack of patient cooperation, unconsciousness, or because stroke was not suspected by the ED teams, but stroke was still suspected by EMSs, stroke protocols were eventually activated in the ED for 25 (3.4%) of them. The EMSs notified the ED about the suspected stroke patients in 587 (79%) cases to activate ED stroke protocols. There were 88 (11.84%) cases where EMSs did not suspect a stroke, but a stroke was diagnosed by the ED.

In the ED, ischemic stroke was diagnosed in 237 (1%) of all patients who arrived with EMSs, hemorrhagic stroke was diagnosed in 21 (0.1%) of all patients who arrived with EMSs, and TIA was diagnosed in 77 (0.3%) of all patients who arrived with EMSs. EMSs calls in which stroke was suspected were grouped by responder type (ALS, BLS, or both) and were compared with the ED diagnoses of stroke (Table 1).

No significant differences were found when comparing accuracy of stroke diagnosis between ages, sexes, different weekdays, weekday vs. weekend, or times of day (*p* > 0.05).

A further analysis was conducted to characterize calls in which a stroke was missed by the EMSs. The ED diagnostic categories for these cases are depicted in Table 2, and the EMSs diagnostic categories for these cases are depicted in Table 3. Of these cases, only in one instance was there a FAST-ED test conducted, in contrast with the other 87 cases.

## 4. Discussion

This was the first study conducted in Israel that aimed to assess the justification of prehospital activation of ED Stroke protocol by comparing EMSs prehospital diagnosis of suspected stroke with the ED diagnoses of stroke or stroke equivalents. This research does not address timeframes, as the study was conducted in a medical center located in central Israel, where ambulance transport times are almost universally under 20 min. In cases of suspected stroke, ambulance evacuation is always urgent, accompanied by lights and sirens.

The aim of this research is not to assess the final diagnosis of stroke but rather to evaluate the activation of the ED stroke protocol by MDA teams. In suspected stroke cases, the ED physician is responsible for the initial assessment and for determining whether the patient should be managed and treated according to the stroke protocol; thus, ED diagnosis of stroke or stroke equivalents was used as the gold standard to determine the justification of activating the ED stroke protocol by EMSs, as in these cases the ED physician would have activated the stroke protocols either way [17]. In a systematic review of EMSs stroke protocols, the importance of pre-stroke diagnosis and its effect on tPA infusion time was examined. It was shown that when the EMSs notified the hospital that a patient may be experiencing a stroke, stroke protocol in the hospital was enacted, and tPA infusion time was decreased, potentially decreasing morbidity and mortality among stroke patients [25].

This study showed that the metrics for MDA as compared with prior studies assessing EMSs activation of ED stroke protocols, had similar sensitivity (63%). However, the PPV (20%) was lower, meaning there are many cases falsely diagnosed as stroke by the Israeli EMSs. ALS units consistently had higher values than when compared to the BLS units. This can be explained by the additional training that the paramedics in the ALS units underwent. The low PPV can be partially attributed to cases when another neurological disorder presented itself and caused EMS providers to mistakenly diagnose the patient with a stroke. In these instances, the index of suspicion should be high, and EMSs should still activate the stroke protocol. However, to effectively use the resources while keeping the NPV high (100%), the ED implemented a rule that when a patient arrives at the ED, the emergency physician will immediately evaluate whether the patient is experiencing a stroke or a different neurological disorder. While the low PPV implicates a very low threshold for ED stroke protocol activation, the goal of identifying every stroke patient is of higher importance than monetary considerations. The most important aspect of the EMSs activating stroke protocol is the preparation of the emergency medicine physician and nurse in the ED to immediately assess the patient and decide about stroke protocol activation “at the doorstep”. This ensures that almost no resources are wasted while also ensuring that nearly all stroke patients are treated as quickly as possible.

The EMSs records show that only 82.22% of patients who were suspected of experiencing a stroke received a FAST-ED exam. The lack of this exam for the remaining 17.76% of patients can be attributed to a few factors; firstly, some of these patients may have been showing non-classic stroke signs that did not fit the FAST-ED criteria. Secondly, some of these patients may not have been cooperative regarding a physical exam or were unconscious. Lastly, patients may have experienced a TIA with symptoms that resolved before the ambulance arrived.

The data also showed that in 21.13% of incidents where the EMSs suspected a stroke, the hospital did not receive notice. There are several possible reasons for this to have occurred: A short evacuation distance from the scene to the hospital, which did not allow enough time for EMSs to alert the ED, the time window for treatment had passed, or the EMS dispatch was not able to contact the hospital in time due to a high workload in the ED office. The possibility of the ED having a high workload is especially possible since Israeli expenditure on healthcare is low compared to other OECD countries [17,21]. Additionally, both human and other healthcare resources are low, which leads to overcrowded EDs and causes difficulty in operative management. These factors further lead to increased morbidity and mortality [22].

Research has shown that optimal in-hospital stroke triage and coordination between the various teams can significantly decrease the time to treatment and improve stroke outcomes [26]. Furthermore, the importance of effective prehospital stroke screening and triage has been shown to decrease the time from stroke onset to endovascular thrombectomy without delaying the administration of IVT and improving patients’ likelihood of recovery [25].

However, a general study on EMS triage in seven regions in the USA showed that 34.3% of triaged emergency patients were low risk. The over-triage in these regions costs the healthcare system a total of 136.7 million dollars annually [27]. Another critique of the overdiagnosis of stroke is that it may result in the further overloading of a hospital’s stroke unit, resulting in inappropriate usage of resources [25]. Implementing a successful stroke triage presents two significant challenges: underdiagnosis would increase patient mortality and overdiagnosis would cause resource and monetary strain.

Since our study is retrospective and the EMS organization uses the standard FAST scale, which we followed, it is possible that employing a more sensitive or specific tool, such as RACE, G-FAST, and CG-FAST [28], could enhance stroke detection. However, this should be examined in future studies to determine whether these tools outperform FAST in our specific EMS setting.

## 5. Limitations

This study has several limitations. First, the data were not stratified by time of day, which may have influenced recognition patterns and outcomes. Additionally, information on the EMS personnel’s years of experience was not available, which could potentially affect the accuracy of stroke identification. Another limitation of the study is the exclusion of patients who left the ED without notice. This group may have included stroke patients who were not properly triaged, potentially introducing a bias and underestimating the number of true stroke cases. Finally, data on stroke severity were also unavailable, limiting our ability to correlate prehospital recognition with clinical outcomes.

## 6. Conclusions

This study showed that while Israeli EMSs over-activate the ED stroke protocol, stroke patients are rarely missed, achieving the main goal of prehospital stroke screening. Additionally, when the EMSs alerts the hospital of a stroke patient, the stroke protocol should be activated and an emergency medicine physician should be ready to immediately assess the patient upon arrival. Increased communication between all levels of the stroke protocol should also be increased to lower the time to treatment. For this reason, multi-level training for physicians, paramedics, and nurses was initiated at SMC in cooperation with MDA and the Shamir Nursing School. To raise the PPV of the EMSs FAST-ED screening, the increased training of EMSs teams in identifying strokes and stroke mimics can help responders to further understand the presenting signs of strokes and differentiate them from other types of neurologic diseases. Additionally, while this study focused on the identification of strokes in patients with symptoms upon their arrival, future research should investigate strokes that develop during patients’ hospital stays.

## Figures and Tables

**Table 1:** Comparison of prehospital diagnosis of stroke with emergency department diagnoses by type of EMS response vehicle.

EMS Response	EMS Diagnosis	Emergency Department Diagnosis		*p* Value	Sensitivity (CI **)	Specificity (CI)	PPV (CI)	NPV (CI)
		Stroke *	Not Stroke					
**BLS Ambulance**	Stroke	111	468	<0.01	61% (54–68)	97% (97–97)	19% (16–22)	100% (99–100)
	Not Stroke	71	14,769					
**ALS Ambulance**	Stroke	24	81	<0.01	73% (56–89)	98% (98–99)	23% (14–31)	100% (100–100)
	Not Stroke	9	5254					
**Both Responses**	Stroke	14	45	<0.01	64% (41–86)	98% (97–99)	24% (12–35)	100% (99–100)
	Not Stroke	8	2207					
**Total**	Stroke	149	594	<0.01	63% (57–69)	97% (97–98)	20% (17–23)	100% (100–100)
	Not Stroke	88	22,230					

* Stroke and stroke equivalents. ** CI—Confidence Interval.

**Table 2:** ED diagnosis categories in cases that were missed by EMSs.

ED Diagnosis—Categories	N
**Hemorrhagic Stroke**	10
**Intracranial Hemorrhage**	8
**Ischemic Stroke**	35
**Other Neurologic**	1
**Transient Ischemic Attack**	34

**Table 3:** EMS diagnosis categories in cases that were missed by EMSs.

MDA Diagnosis—Categories *	N
**Cardiac**	6
**Other**	21
**Other Neuro**	58
**Trauma**	12
**Toxicologic**	2
**Vascular**	8

* Each case may have more than one EMS diagnosis and thus more than one category.

## Data Availability

Raw data were generated at Shamir Medical Center. Data supporting the findings of this study are available from the corresponding author on request.

## Abbreviations

The following abbreviations are used in this manuscript:

tPA	Tissue plasminogen activator
EMSs	Emergency medical services
ED	Emergency department
TIA	Transient ischemic attack
NPV	Negative predictive value
PPV	Positive predictive value
SBP	Systolic blood pressure
DM	Diabetes mellitus
FAST-ED	Field assessment stroke triage for emergency destination
RACE	Rapid arterial occlusion evaluation
LAPSS	Los Angeles prehospital stroke screening
PreHAST	PreHospital ambulance stroke test
MDA	Magen David Adom
SMC	Shamir medical center
BLS	Basic life support
ALS	Advanced life support
EMT	Emergency medical technician
RECORD	Reporting of studies conducted using observational routinely collected health data
STARD	Standards for reporting diagnostic accuracy
IRB	Institutional review board
CI	Confidence interval
